# A simulation study comparing the power of nine tests of the treatment effect in randomized controlled trials with a time-to-event outcome

**DOI:** 10.1186/s13063-020-4153-2

**Published:** 2020-04-06

**Authors:** Patrick Royston, Mahesh K. B. Parmar

**Affiliations:** MRC Clinical Trials Unit at UCL, Institute of Clinical Trials and Methodology, 90 High Holborn, London, WC1V 6LJ UK

**Keywords:** Randomized controlled trials, Time-to-event outcome, Logrank test, Hazard ratio, Non-proportional hazards, Versatile test, Power, Simulation, Robustness

## Abstract

**Background:**

The logrank test is routinely applied to design and analyse randomized controlled trials (RCTs) with time-to-event outcomes. Sample size and power calculations assume the treatment effect follows proportional hazards (PH). If the PH assumption is false, power is reduced and interpretation of the hazard ratio (HR) as the estimated treatment effect is compromised. Using statistical simulation, we investigated the type 1 error and power of the logrank (LR)test and eight alternatives. We aimed to identify test(s) that improve power with three types of non-proportional hazards (non-PH): early, late or near-PH treatment effects.

**Methods:**

We investigated weighted logrank tests (early, LRE; late, LRL), the supremum logrank test (SupLR) and composite tests (joint, J; combined, C; weighted combined, WC; versatile and modified versatile weighted logrank, VWLR, VWLR2) with two or more components. Weighted logrank tests are intended to be sensitive to particular non-PH patterns. Composite tests attempt to improve power across a wider range of non-PH patterns. Using extensive simulations based on real trials, we studied test size and power under PH and under simple departures from PH comprising pointwise constant HRs with a single change point at various follow-up times. We systematically investigated the influence of high or low control-arm event rates on power.

**Results:**

With no preconceived type of treatment effect, the preferred test is VWLR2. Expecting an early effect, tests with acceptable power are SupLR, C, VWLR2, J, LRE and WC. Expecting a late effect, acceptable tests are LRL, VWLR, VWLR2, WC and J. Under near-PH, acceptable tests are LR, LRE, VWLR, C, VWLR2 and SupLR. Type 1 error was well controlled for all tests, showing only minor deviations from the nominal 5%. The location of the HR change point relative to the cumulative proportion of control-arm events considerably affected power.

**Conclusions:**

Assuming ignorance of the likely treatment effect, the best choice is VWLR2. Several non-standard tests performed well when the correct type of treatment effect was assumed. A low control-arm event rate reduced the power of weighted logrank tests targeting early effects. Test size was generally well controlled. Further investigation of test characteristics with different types of non-proportional hazards of the treatment effect is warranted.

## Background

Randomized controlled trials (RCTs) with a time-to-event outcome are typically designed according to sample size and power calculations using the logrank test. The treatment effect is summarized by the hazard ratio (HR) between the control and research arms, usually estimated with a Cox proportional hazards (PH) model. During the last decade or so, researchers e.g. [1, 2] have demonstrated that non-proportional hazards (non-PH) occur fairly often in trials across a range of medical research areas. Non-PH may threaten the power of the logrank test, potentially distorting the findings of a trial and jeopardizing its success. It is therefore important that trial designers take into account the possibility and, if feasible, the probable nature of non-PH in the particular setting of the study.

We assume that HR <1 denotes a reduction in the hazard of an event (e.g. death) in a research arm. Non-PH means that the HR varies systematically over follow-up time. We may usefully distinguish four types of HR patterns: PH, early or diminishing effect, late or delayed effect, other. PH includes the null-hypothesis case of identical survival curves in the trial arms. With an early effect, the HR is <1 in the early follow-up and increases later. An early effect may, for example, be provoked by ‘wearing off’ of the effectiveness of a therapy that is administered for a limited period and then stopped. A late or delayed effect may occur in screening or prevention trials or in trials in immuno-oncology settings, in which the treatment effect is expected to take time to manifest. Subsequently, we refer to such patterns generically as late effects.

The ‘other’ type covers all other possibilities, of which the most readily recognizable are crossing survival functions. Our impression is that in real trials, PH, early and late patterns predominate. Other patterns are not necessarily simple to characterize; therefore, only these three are studied in the present paper.

**Fig. 1:**
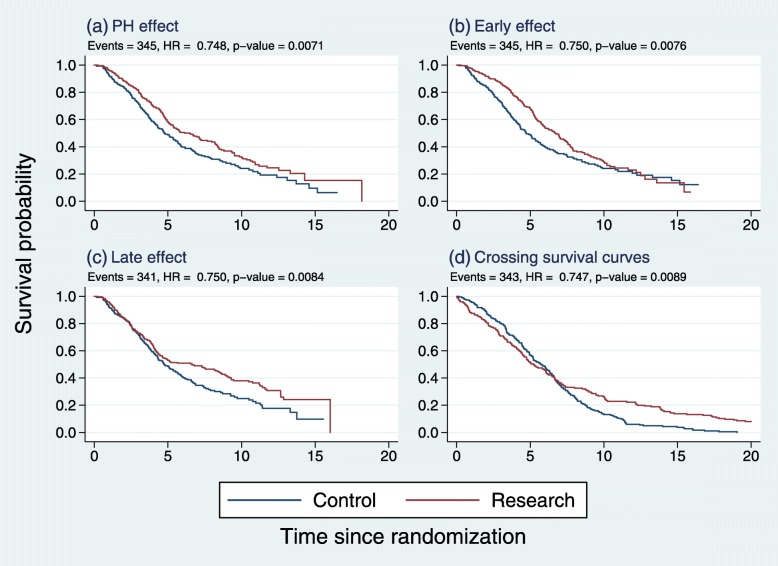
Kaplan-Meier survival curves in simulated datasets with similar HRs and *p* values, but with four types of treatment effects: **a** PH, **b** early, **c** late, **d** other (crossing survival curves)

Figure 1 gives examples of pairs of Kaplan-Meier survival curves illustrating the four types of treatment effects we have discussed.

We created the datasets by simulation to illustrate reasonable and plausible curves. Although the overall HRs, numbers of events and logrank test *p* values for the treatment comparisons are approximately the same in each case, the survival-curve comparisons differ considerably. For example, the difference in median survival time (research minus control) is largest with the early and late effects, somewhat smaller with PH and close to zero (and negative) for the ‘other’ pattern (d).

The focus of the present paper is on tests of the null hypothesis of identical survival functions against specific alternative hypotheses (PH, early effect, late effect). It is widely recognized that the logrank test may lose power, sometimes severely, in non-PH situations. Making extensive use of simulation, our aim is to identify good candidate(s) for resilience from a set of nine tests that we have selected. A ‘resilient’ test is one that exhibits acceptable power under PH and also under some common patterns of non-PH, while maintaining the type 1 error rate close to the nominal level. In addition to power, we therefore also assessed the type 1 error of the tests. The tests and the rationale for their selection are described in the next section.

The structure of the article is as follows. In ‘Methods’, we describe the tests to be compared and our approach to simulation of the performance (power) of the tests. In ‘Results’ we report our findings on type 1 error and power. This section also includes a comparison of the tests on three selected trials with apparently differing types of treatment effects. We end with a Discussion and our Conclusions.

## Methods

### Tests to be compared

Many tests of two survival curves have been proposed over the last five decades or so, but very few have found their way into practice in trials. We have focused on the most popular, the logrank, a small number of related tests and particularly on more recent composite tests comprising two or more component tests. The logrank test is the *de facto* standard for trial design and analysis and is therefore the natural comparator for other tests. Variants of the logrank test are typically weighted in such a way as to be sensitive to particular non-PH patterns. Composite tests are an attempt to improve power across a wider range of non-PH patterns than the logrank test manages.

We have not included tests which require prespecifying a single, fixed time point, *t*^∗^ say, for their evaluation. Examples are the difference at *t*^∗^ in Kaplan-Meier survival estimates or in restricted mean survival times (RMSTs). Although such tests are intuitively simple and appealing, their power with some non-PH patterns is vulnerable to poor choices of *t*^∗^.

The nine tests we have included are described briefly below. All computations were performed using Stata 15.1 [3].

#### Logrank test (LR)

The logrank test is the optimal (most powerful) rank test under PH. The test is also valid under non-PH alternatives, but it may then lack power.

#### Early-effect weighted logrank test (LRE)

LRE is a weighted logrank test with Fleming-Harrington weight function (1,0) [4]; that is *w*_*i*_=*S*(*t*_*i*_−0). LRE is intended to be sensitive to early effects. It is similar to the Peto-Peto-Prentice test [5, 6].

#### Late-effect weighted logrank test (LRL)

LRL is a weighted logrank test with Fleming-Harrington weight function (0,1); that is *w*_*i*_=1−*S*(*t*_*i*_−0). LRL is intended to be sensitive to late effects.

#### Supremum logrank test (SupLR)

The supremum logrank test [7] is based on the maximal logrank test statistic over the event times *t*_1_,…,*t*_*r*_. It is calculated by restricting the logrank test to time *t*_*i*_ and then taking the supremum test statistic over the *t*_*i*_. Local minima or maxima in the test statistic may be detected by the supremum logrank test which may indicate a non-random difference between the survival functions.

#### Joint test (J)

The joint test [8] combines a Cox test (essentially identical to the logrank test) with a standard test of non-PH, the Grambsch-Therneau test. Under PH, the two component tests are independent. The joint test statistic is the sum of the two model chi-square values. It has a known distribution under the null and under PH alternatives.

#### Combined test (C)

The combined test [9] combines a Cox test with a permutation test based on the maximal squared standardized difference in RMST between the control and research arms. Maximization is over a predefined small set of event times (*t*^∗^). Royston et al. [2] showed that the combined test outperformed the Cox test (and implicitly the logrank test) in 55 randomized comparisons based on reconstructed data from 50 RCTs in various medical research areas.

#### Weighted combined test (WC)

The weighted combined test (unpublished, available in Stata from the first author) is an attempt to improve the performance of the combined test when a delayed/late treatment effect is present. The Cox test component is replaced by the LRL test.

#### Versatile weighted logrank test (VWLR)

A ‘versatile’ test is one derived by combining several weighted logrank tests in different ways [10, 11]. Like the C and WC tests, a versatile test is designed to be sensitive to different types of departures from the null hypothesis *H*_0_:*S*_0_(*t*)=*S*_1_(*t*). Karrison’s proposal [12] which we use here, is the maximum square-root chi-square statistic among three correlated logrank tests: unweighted (i.e. standard LR), early-effect weights (as in LRE) and late-effect weights (as in LRL). The asymptotic null distribution of the test statistic is available in closed form.

#### Modified versatile weighted logrank test (VWLR2)

VWLR2, the modified version of VWLR, is unpublished and is available as a Stata program from the first author. It incorporates a small but potentially important change to one component of the VWLR test. The LRE test with weights *w*_*i*_=*S*(*t*_*i*_−0) is replaced by a logrank test with weights given by

$$ w_{i}=\max\left\{0.001,\left[ S\left(t_{i}-0\right) -S_{\min}\right] /\left(S_{\max}-S_{\min}\right) \right\}   $$

where *S*_max_=1 and *S*_min_ is the minimum of the left estimate, *S*(*t*−0), of the Kaplan-Meier survival function. The support of these weights is the interval [0.001,1]. The aim is to increase power when the data exhibit an early effect with a low event rate. With such data, the coefficient of variation of the original weights is small, and therefore the weighted test (LRE) too closely resembles the standard test (LR). By construction, the issue of too-similar weights does not arise with the LRL component of VWLR. The null distribution of the test statistic for VWLR2 follows from the general result for the maximum of weighted logrank tests as derived in [4], section 7.5, theorem 7.5.1.

### Simulation scenarios

We assessed the power of the nine tests under four alternatives: null case (identical survival distributions in control and research arms), PH with HR =0.75, early effect, late effect. Furthermore, we studied two survival distributions in the control arm: high event rate (*S*_min_≃0.1) and low event rate (*S*_min_≃0.9). The goal was to identify, within the constraints of the simulation design, the test(s) which performed best under PH, early effect, late effect and overall across all three patterns.

### Approach to simulation

#### Survival distributions

**Fig. 2:**
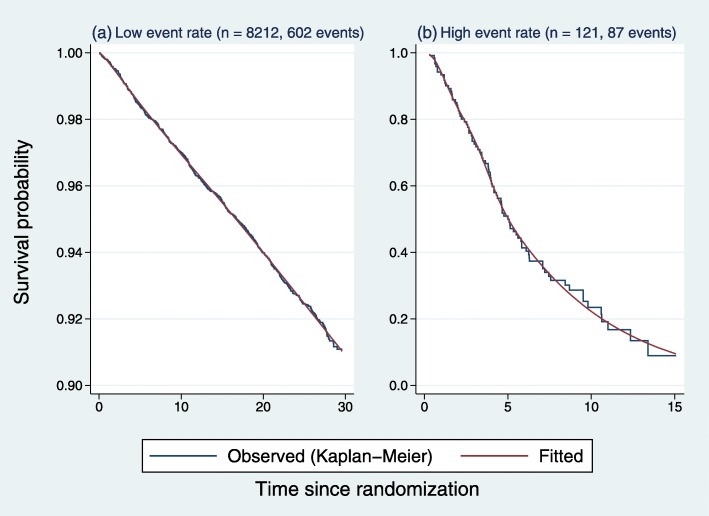
Survival functions in the control arm of two real trials used as the basis of simulations: **a** low event rate, **b** high event rate. *Jagged lines*, Kaplan-Meier estimates; *smooth lines*, estimates from flexible parametric models with 5 degrees of freedom. Note the different scaling of the vertical axes

Survival distributions in the control arms of two real trials were chosen to represent the survival function in the control arm of simulated datasets with low or high event rates, respectively. The first trial [13] had a low event rate and the second [14] a high event rate. The survival functions were approximated using flexible parametric models [15, 16]. A restricted cubic spline with 5 degrees of freedom was used to model the log cumulative hazard function as a function of log time to event in each control arm. Figure 2 shows the observed (Kaplan-Meier) and fitted survival functions in each of the original datasets.

We used the same approach to approximate the distribution of time to censoring in each dataset (data not shown).

From the fitted flexible parametric models, we obtained small numbers of parameters which describe the survival and time-to-censoring distributions in the two datasets. We used the estimated parameter values with suitable sample sizes in subsequent simulations by applying the community-contributed Stata package stsurvsim [17, 18]. Further details are given below.

#### Simulating early and late treatment effects

To create simple early and late treatment effects, we chose step functions for the time-dependent hazard ratio, HR (*t*), as shown in Table 1.

**Table 1:** Hazard ratios defining treatment effects with non-proportional hazards in the simulation studies

Effect	Event	HR (*t*)	
type	rate	*t*≤*t*^∗^	*t*>*t*^∗^
Early	Low	0.3	1.0
	High	0.5	1.0
Late	Low	1.0	0.3
	High	1.0	0.5

The design implements a treatment effect (expressed as HR) that persists over (0,*t*^∗^) and then ceases (early effect), or one that is 1.0 when *t*≤*t*^∗^ and <1.0 for *t*>*t*^∗^ (late effect).

If the change point *t*^∗^ is ‘large’ in the early-effect case, the HR will be nearly constant over the observed follow-up, and the treatment effect will be close to PH. Vice versa, if *t*^∗^ is ‘close to 0’ in the late-effect case, the treatment effect will be close to PH. We expect the logrank test to perform (nearly) optimally in such a situation. In other situations, we would expect tests specifically designed to detect types of non-PH to outperform the LR.

The time scale embodied in *t*^∗^ is arbitrary. A less scale-dependent meaning of *t*^∗^ being ‘large’ and ‘close to 0’ may be attributed to the cumulative proportion of events in the trial before *t*^∗^, known as the information fraction (IF). The IF is an important parameter of the alpha-spending functions for group-sequential trials. To remove the effect on the IF of the alternative distributions we simulated, we limited the IF to the control arm.

We quantified the performance of the nine tests in the early- and late-effects cases in relation to the control arm IF as follows. We selected seven suitably placed values of *t*^∗^ for each effect type and event rate (see Table 2).

**Table 2:** Time points (*t*^∗^) used in the simulation studies

Event rate		Early effect	Late effect
Low	*t*^∗^	10 12 14 17 19 22 25	0 4 7 10 13 16 19
	IF%	40 49 56 68 75 87 95	0 16 28 40 53 64 75
High	*t*^∗^	3 4 5 6 7.5 9 10.5	0 1 2 3 4 5 6
	IF%	45 57 69 77 85 91 96	0 12 27 45 57 69 77

IF% denotes the information fraction expressed as a percentage of the total number of events in the control arm of the original datasets. See text for further details

The *t*^∗^ values given in Table 2 provide an appropriate spread of IFs in the control arm of the two original datasets.

With each chosen value of *t*^∗^, we computed the sample size for each of the four designs (low/high event rate by early/late effect) using the Analysis of Resources for Trials (ART) community-contributed software package for Stata [19]. The procedure was used to obtain sample sizes that offer a power of 80% or 90% for the LR. We took the LR as the benchmark test for power comparisons with the other eight tests. Based on the benchmark power, the sample sizes used in the simulations varied widely, between about 100 and 18,000 (data not shown).

**Fig. 3:**
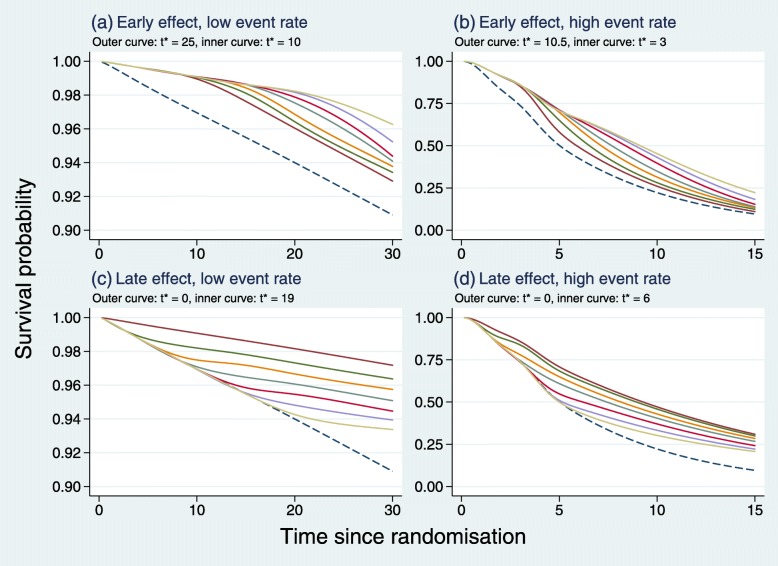
True survival functions used in the simulations. *Dashed lines* represent the control arm, *other lines* the research arm with the change point in the HR at different values of *t*^∗^

The ‘true’ survival functions used in the simulations for each of the scenarios shown in Table 2 are illustrated in Fig. 3.

Note that in panels c and d of Fig. 3, *t*^∗^=0 corresponds to PH. Despite the abrupt, seemingly unrealistic step changes in the HR at *t*^∗^ used to define non-PH, the survival functions look both reasonable and plausible.

Realistic datasets were simulated for each arm of a hypothetical trial with 1:1 treatment allocation using the flexible parameter estimates and the Stata program stsurvsim cited in the previous section. Five thousand replicates were simulated for each power, event rate, effect type and value of *t*^∗^. Power of a given test at the two-sided 5% significance level was estimated as the number of replicates in which *p*<0.05, divided by 5000. Mean observed IF values in the control arm of the simulated datasets were used to define the *x*-axis in graphs of the power for the various tests and conditions.

#### Treatment effects under PH

Treatment effects under PH are covered by the special case of late effects with *t*^∗^=0 and were not handled separately.

#### Type 1 error

Treatment effects under the null hypothesis *H*_0_:*S*_0_(*t*)=*S*_1_(*t*) were tested using simulation, as with the power studies. For each chosen sample size (*n*), we simulated 5000 replicate datasets comprising two replicates each with *n*/2 observations, based on the estimated time-to-event and time-to-censoring distributions in the control arm. We took 12 values of *n* in the range [400,10,000] in the low event-rate scenario and 12 more in the range [40,1000] in the high event-rate scenario. Under PH, the effective sample size is the number of events. With the given sample sizes and event rates, we aimed to cover a wide range of numbers of events.

The empirical type 1 error (size) of a given test at the nominal *α*=0.05 level was estimated as the number of replicates in which *p*<0.05, divided by 5000. A test with size exceeding 0.05 is termed ‘anti-conservative’ or ‘liberal’, whereas one whose size is below 0.05 is deemed ‘conservative’.

## Results

### Type 1 error

We estimated the empirical type 1 error (size) of the nine tests at the *α*=0.05 level by simulation. We pooled the 5000 replicates for each event rate and sample size into datasets, each with 5000×12=60,000 observations. Results by event rate are given in Table 3.

**Table 3:** Empirical type 1 error (size) of the nine tests in 60,000 simulated samples for low and high event rates

Test	Abbrev.	Low event rate		High event rate	
		Size (%)	95% CI	Size (%)	95% CI
Logrank	LR	5.0	(4.8,5.2)	5.1	(4.9,5.3)
Logrank (early)	LRE	5.0	(4.8,5.2)	5.1	(5.0,5.3)
Logrank (late)	LRL	4.9	(4.7,5.1)	5.3	(5.1,5.4)
Supremum logrank	SupLR	**4.5**	(**4.4,4.7**)	**4.6**	(**4.5,4.8**)
Joint	J	5.1	(4.9,5.3)	5.0	(4.9,5.2)
Combined	C	4.9	(4.7,5.1)	5.0	(4.8,5.2)
Weighted combined	WC	4.8	(4.6,5.0)	5.4	(5.2,5.6)
Versatile WLR	VWLR	5.0	(4.8,5.2)	5.2	(5.0,5.4)
Versatile WLR (modified)	VWLR2	5.1	(4.8,5.1)	5.2	(5.0,5.4)

The supremum logrank (SupLR, results shown in bold type) test stands out as it is conservative for both event rates, the size being about 4.5*%*. For the low event rate, the size of the remaining tests is close to the nominal 5%, whereas for the high event rate, the WC, LRE, LRL, VWLR and VWLR2 tests appear a little anti-conservative.

Further investigation (data not shown) revealed that minor size inflation may occur in the high event-rate case when there are fewer than approximately 100 events in the dataset (see Table 4).

**Table 4:** Empirical type 1 error (size) of the nine tests in pooled simulated samples with low or high event rates and ≤100 events in each simulation replicate

Test	Abbrev-	Low event rate		High event rate	
	iation	Size	95% CI	Size	95% CI
Logrank	LR	5.2	(4.9,5.6)	5.5	(5.1,5.8)
Logrank (early)	LRE	5.2	(4.9,5.6)	5.3	(4.9,5.6)
Logrank (late)	LRL	4.8	(4.5,5.2)	6.0	(5.6,6.3)
Supremum logrank	SupLR	4.3	(4.0,4.6)	4.5	(4.2,4.9)
Joint	J	5.2	(4.9,5.6)	5.1	(4.8,5.5)
Combined	C	5.1	(4.7,5.4)	5.6	(5.2,5.9)
Weighted combined	WC	4.7	(4.4,5.0)	6.2	(5.8,6.6)
Versatile WLR	VWLR	5.1	(4.8,5.4)	5.7	(5.3,6.1)
Versatile WLR (modified)	VWLR2	5.0	(4.7,5.4)	5.7	(5.4,6.1)

Pooled sample sizes of simulated datasets are 15,818 and 15,527, respectively

The SupLR test is again conservative for both event rates. Inflation of the size of most of the other tests, including the LR test, occurs with the high event rate. Such inflation is not evident when there are more than 100 events (data not shown).

Aside from minor issues, all of the empirical type 1 errors of the nine tests are close to the nominal, two-sided 5% significance level. We are therefore justified in performing power assessments of all the tests.

### Power

We report power results for an early effect and then for a late effect. Each category is subdivided into low and high event rates.

#### Early effect

##### Low event rate

**Fig. 4:**
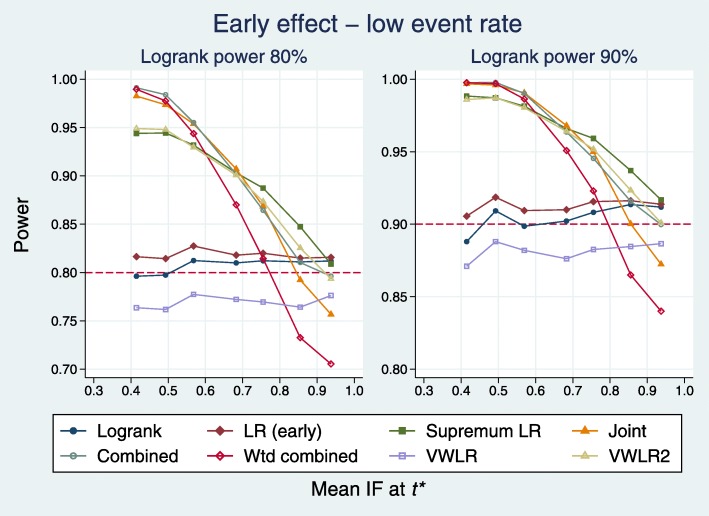
Power of eight tests versus mean IF at *t*^∗^ for early effects with a low event rate. *t*^∗^ denotes the time point of the change in the hazard ratio

Figure 4 shows the power for eight tests as a function of the mean IF.

We have excluded the LRL test because its power is low in this setting and its results reduce the legibility of the plots. When IF <0.8, five tests are superior to the logrank: J, C, WC, SupLR and VWLR2. When IF >0.8, the treatment effect approaches PH. Here, the WC, J and VWLR tests are weakest; the other five tests perform about the same. Irrespective of the IF, the LRE test is slightly better than the LR.

**Fig. 5:**
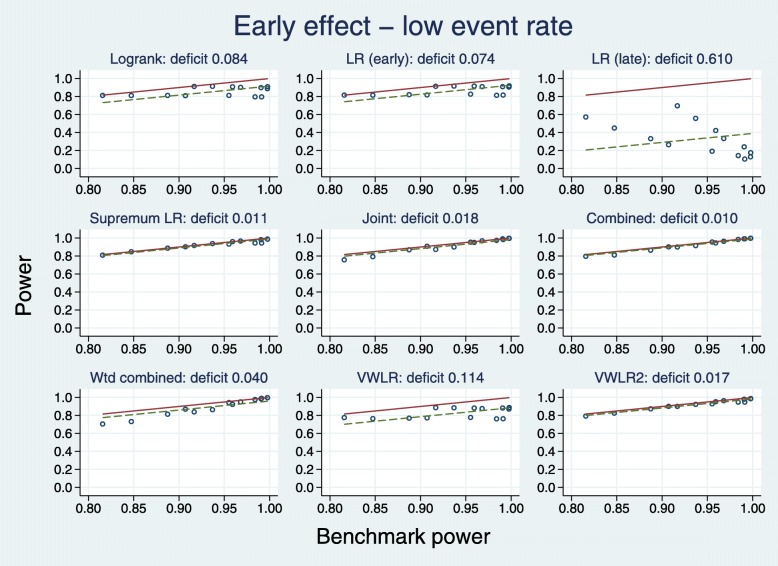
Test power compared with benchmark for early effects with a low event rate

Figure 5 displays the results in a different way. For each of the 7+7=14 sets of simulated datasets, the test with the largest empirical power is identified. This ‘best’ result among all nine tests serves as the benchmark power and constitutes the horizontal axis of each plot. Note that the test which performs best is not necessarily the same for all 14 sets of datasets.

For a given test, we define the deficit to be the mean difference over the 14 datasets between the power of the test and the benchmark. The deficit for each test is plotted as the dashed horizontal line parallel to the solid line of identity representing the benchmark.

According to the deficit metric, the five best tests (with the deficit in parentheses) are C (0.010), SupLR (0.011), VWLR2 (0.017), J (0.018) and WC (0.040). The LRL test is by far the worst performer here (deficit 0.610).

##### High event rate

Plots for the high event rate equivalent to Figs. 4 and 5 are 6 and 7, respectively.

**Fig. 6:**
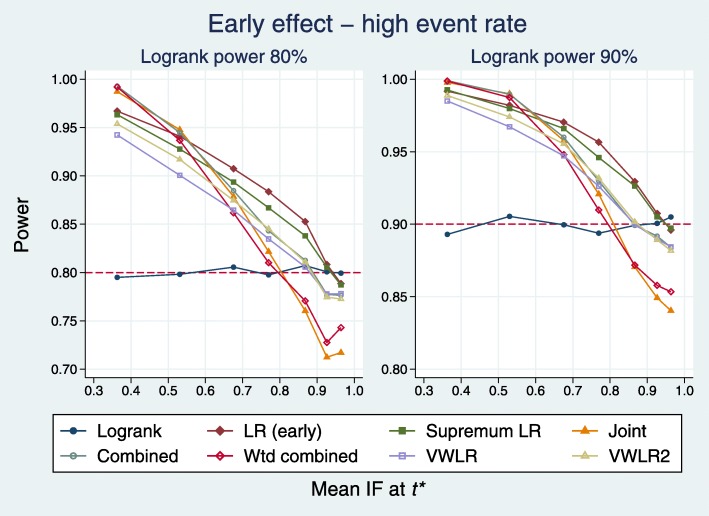
Power of eight tests versus mean IF at *t*^∗^ for early effects with a high event rate

**Fig. 7:**
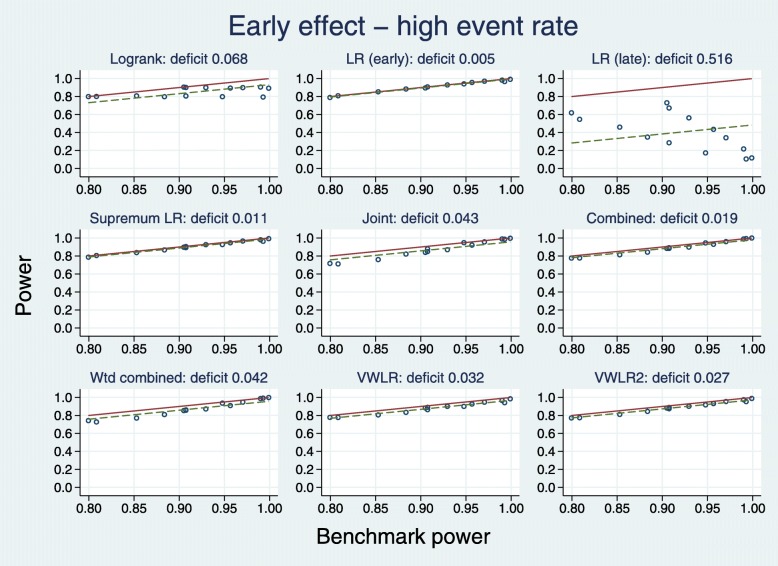
Test power compared with benchmark for early effects with a high event rate

A slightly different pattern emerges. With IF <0.8, all tests (except LRL, excluded) outperform the LR. With IF >0.8, two tests are worst: J and WC.

All tests except LRL have broadly similar deficits, with that for the LR test being the largest (0.068). The VWLR and VWLR2 tests now perform about the same.

#### Late effect

##### Low event rate

Plots equivalent to Figs. 4 and 5 are 8 and 9.

**Fig. 8:**
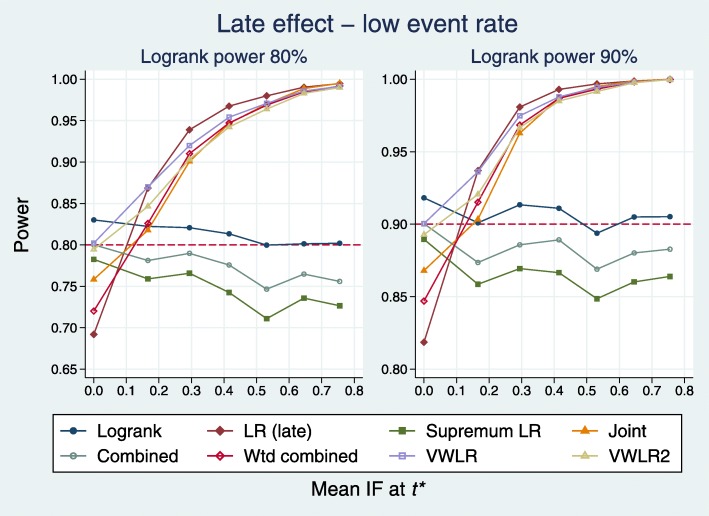
Power of eight tests versus mean IF for late effects with a low event rate

**Fig. 9:**
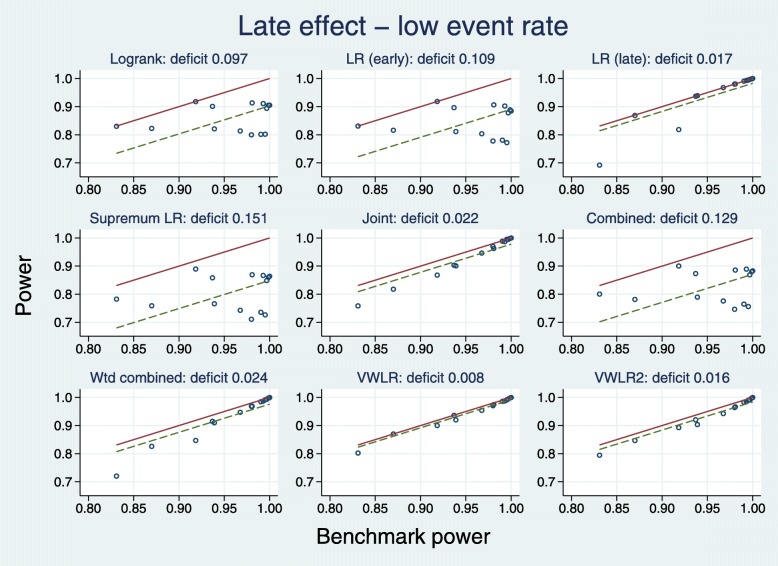
Test power compared with benchmark for late effects with a low event rate

When IF >0.2, five tests dominate: J, WC, LRL, VWLR, VWLR2. The C and SupLR tests are consistently worse than LR. When IF <0.2 (near PH), the worst three tests are J, WC, LRL.

The deficits show a clear picture. The best five tests are J (0.022), WC (0.024), LRL (0.017), VWLR (0.008), J (0.016). The C (0.129), SupLR (0.151) and LRE (0.109) tests are all worse than the LR (0.097).

##### High event rate

Plots for the high event rate equivalent to Figs. 8 and 9 are 10 and 11, respectively.

**Fig. 10:**
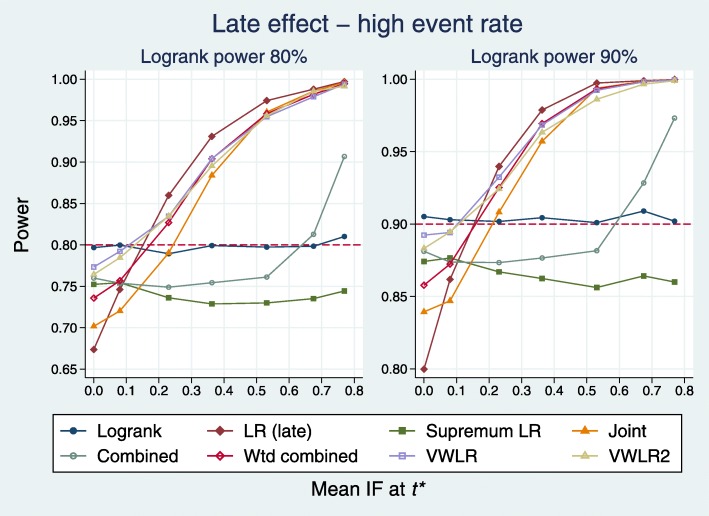
Power of eight tests versus mean IF at *t*^∗^ for late effects with a high event rate

**Fig. 11:**
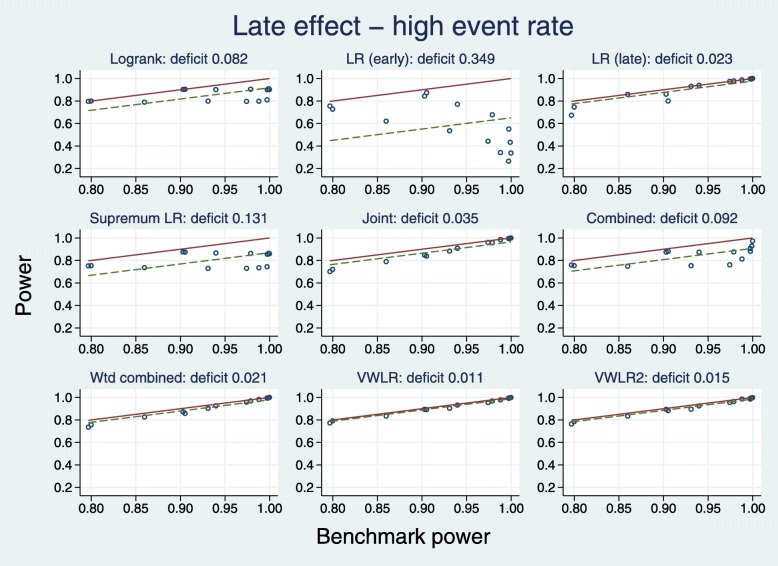
Test power compared with benchmark for late effects with a high event rate

The results are broadly similar to those for the low event rate.

#### Summary

**Fig. 12:**
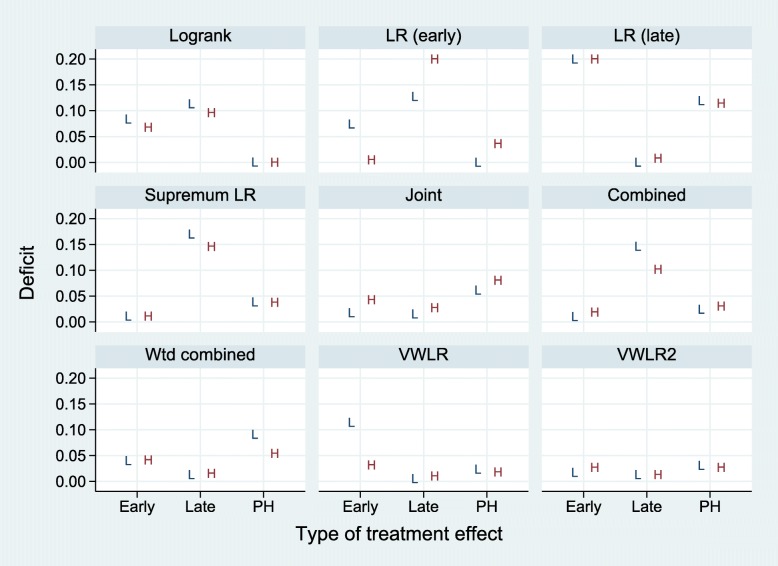
Summary of power results for nine tests and three types of treatment effects (early, late, PH) and two event rates (low (L) and high (H)). Values for LR (early) and LR (late) tests plotted at 0.2 indicate deficits of 0.2 or more

The power results for the four subcases (early/late effects by low/high event rates) are summarized in Fig. 12.

Results for PH treatment effects (represented by late effects with *t*^∗^=0) have been extracted separately, and deficits for late effects exclude them. For a given type of treatment effect, we subjectively defined as acceptable a test with a mean deficit across low and high event rates of <0.05. Acceptable tests and their mean deficits are summarized in Table 5.

**Table 5:** Summary of findings from our simulation studies

Effect	Acceptable tests (mean deficits over low and high event rates)
Early	SupLR (0.011), C (0.015), VWLR2 (0.022), J (0.030), LRE (0.040), WC (0.041)
Late	LRL (0.004), VWLR (0.008), VWLR2 (0.013), WC (0.014), J (0.021)
PH	LR (0.000), LRE (0.018), VWLR (0.021), C (0.027), VWLR2 (0.029), SupLR (0.038)

Acceptable tests for different presumed types of treatment effects in increasing order of their mean power deficits. See text for details

If an early effect is expected, acceptable tests are SupLR, C, VWLR2, J, LRE and WC. If a late effect is expected, acceptable tests are LRL, VWLR, VWLR2, WC and J. Under PH, acceptable tests are LR, LRE, VWLR, C, VWLR2 and SupLR.

If the expected type of treatment effect is unknown, our preference is for VWLR2, since it is the only test that is acceptable with all three types of treatment effects. Its maximum deficit of 0.031 across the six individual results is the smallest among the nine tests and the only one that is <0.05. VWLR2 also has the smallest overall mean deficit (0.021). On this criterion the second-best test is J, with a maximum deficit of 0.080 (mean 0.041). However, J performs poorly under PH. See also Fig. 12.

### Example

We exemplify the performance of the nine tests with three RCTs, chosen because they appear to show early, late and PH treatment effects. PATCH1 [20] concerns treating cellulitis of the leg. UKCTOCS [21] is a trial of screening for ovarian cancer. For illustration, we have combined the two research arms (different screening modes) into a single arm (screenees). RE01 [22] compares palliative treatments in advanced kidney cancer. Table 6 gives basic information on the studies.

**Table 6:** Basic information for the three example trials

	Trial		
	PATCH1	UKCTOCS	RE01
Outcome (time to)	Recurrence	Ovarian cancer death	Death (any cause)
Research arm	Penicillin	Screening	Interferon- *α*
Control arm	Placebo	No screening	MPA
Type of treatment effect	Early	Late	PH
Event rate	Low/medium	Low	High
*n*	274	202,546	347
Events	129	649	322
*S*_min_	0.419	0.996	0.045

**Fig. 13:**
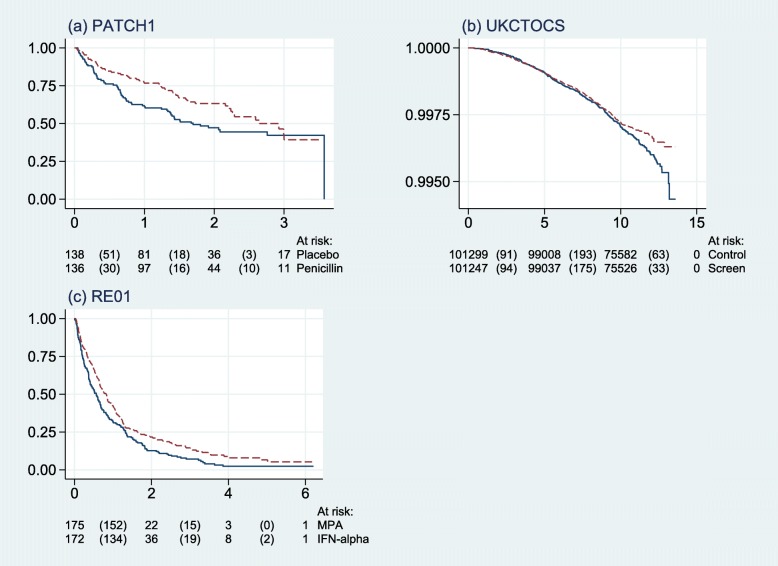
Kaplan-Meier survival curves for the three example trials. *Horizontal axis* shows years since randomization. *Solid lines*, control arm; *dashed lines*, research arm. *Values in parentheses* below the graphs denote number of events in each interval

Figure 13 shows Kaplan-Meier curves for the three datasets.

In Fig. 13 note the large sample size and very low event rate in b, the UKCTOCS ovarian cancer screening trial.

Table 7 shows the *p* values for the treatment effect on applying the nine tests to the trial datasets.

**Table 7:** *p* values for the nine tests on three example datasets

Test	Trial		
	PATCH1	UKCTOCS	RE01
	(early effect)	(late effect)	(PH)
LR	0.052	0.076	**0.009**
LRE	**0.020**	0.077	**0.010**
LRL	0.573	**0.008**	0.047
SupLR	**0.012**	0.153	**0.013**
J	**0.023**	**0.028**	0.028
C	**0.023**	0.112	**0.014**
WC	**0.027**	**0.014**	0.036
VWLR	0.036	**0.013**	**0.018**
VWLR2	**0.017**	**0.016**	**0.019**

Values in bold type indicate acceptable tests according to the simulation results. See text for details

It is striking that, in each dataset, the tests deemed ‘acceptable’ for the corresponding type of treatment effect have the lowest *p* values (shown in bold type) among the nine.

In PATCH1, the ‘standard’ test, LR, just misses significance at the conventional 0.05 level. All other tests except LRL are significant. In UKCTOCS, only the acceptable tests for a late effect are significant. In RE01, all the tests are significant, while LR has the smallest *p* value.

### Are alternative tests ready for the primary analysis?

Based largely on our simulation results, we have recommended VWLR2 as a good choice of resilient test under prior ignorance of the characteristics of the survival curves. However, as with all simulation studies, only a tiny fraction of possible types of survival curves has been explored. For example, a recent editorial in the context of cancer trials [23] (see their Figure A1) demonstrated anomalous behaviour of the LRL test. The authors showed a constructed example in which the experimental arm survival curve always lay below the control-arm curve, whereas the late-effect (LRL) test rejected the null hypothesis in favour of the experimental arm. Such a result seems to contradict common sense. However, it can be understood in terms of the conditional survival distributions that manifest after the initial steep drop in survival in the experimental arm. Details will be explored elsewhere.

For weighted logrank tests and versatile tests that include them, several strands of supporting research evidence are lacking before the tests may be regarded as serious candidates for practical use in trial design and analysis. For example, we need more comprehensive examples of their characteristics under different non-PH patterns, possibly including a more detailed and nuanced understanding of the effect of the Fleming-Harrington parameter values on test power.

For alternative tests in general, we need to know how to use them in the primary analysis, how to assess data maturity (readiness to analyse accrued data) and also how to perform intermediate analyses for benefit or lack of benefit.

We conclude that although useful progress has been made, much needs to be done before any proposed alternative tests are considered ready for the primary analysis of trial data.

## Discussion

Based on our extensive simulation study, we conclude that the modified VWLR test VWLR2 is probably the best general choice among the nine tests we have compared when the form of any difference between survival curves cannot be predicted reliably in advance. We would argue that this would hold true in many cases. The VWLR2 test has an advantage over the combined (C) and weighted combined (WC) tests in that the distribution of the test statistic is known under the null hypothesis *S*_0_(*t*)=*S*_1_(*t*). An important question is whether the simplified type of non-PH we have investigated here (see further remarks below) is general enough to enable a broader recommendation. This question can really only be addressed by both performing further simulation studies with a wider range of alternative hypotheses and comparing the preferred test(s) with others when applied to a varied spectrum of datasets from real RCTs.

In an unpublished research report posted online (see https://arxiv.org/abs/1909.09467v1) after our manuscript was submitted to *Trials*, Lin and colleagues [24] came to conclusions broadly similar to ours. They also used Monte Carlo simulation to study the performance of nine tests, of which only LR, LRL and LRE overlapped our set. The six additional tests were a weighted logrank test with index (1,1); a versatile weighted logrank test with four components (MaxCombo, identical to the present VWLR test except that it also includes the (1,1) test); difference in restricted mean survival time; Breslow’s test; weighted Kaplan-Meier test; and Lee’s combination test. Besides PH and a range of early-effect and late-effect non-PH examples, they investigated scenarios with crossing survival curves. Consistent with us, they summarised by stating ‘There is not a single most powerful test across all scenarios. In the absence of prior knowledge regarding the PH or non-PH patterns, the MaxCombo test is relatively robust across patterns.’ In both our and their investigations, a composite weighted logrank test seems to perform well.

In a recent analysis [2], we compared the combined test (C) with the Cox test (very similar to LR). We found results in favour of the combined test in an analysis of datasets reconstructed from the published Kaplan-Meier survival curves in 50 phase III RCTs. The trials, which were reported in four leading medical journals in 2013, were in a variety of medical research areas. In this particular sample of trials, graphical analysis suggested that significant treatment effects were mostly near-PH or early in nature, clear late effects being rare. However, in some areas of medical research, for example immuno-oncology and screening and prevention trials, late effects are often anticipated. Thus, the potential to detect late effects remains important.

As with all simulation studies, due to the inevitable restriction on the numbers and types of scenarios that may be investigated, interpretation and generalizability of results require caution. We have limited our early and late scenarios to piecewise constant HRs with a single change point placed at different time points (see Fig. 3 for the corresponding survival functions). The full range of possible early or late effects is not and cannot be represented. However, our approach allowed us to study how the position of the change point in the HR in relation to the control-arm information fraction affected power, taking the LR test as the benchmark. This turned out to be an important consideration (see Figs. 4, 6, 8, 10). The five tests we identified as most powerful for an early effect were superior only when the change point was at IF ∼0.8 or smaller. The equivalent condition for detecting a late effect was IF ∼0.2 or larger. Whether such a characterization is of practical help in selecting a test prospectively when designing a new trial needs further exploration.

A major issue we have not considered here is how best to describe and estimate treatment effects under non-PH. Hitherto, standard practice has been to use a test and an estimate of the treatment effect, together with its confidence interval (CI), that are coherent. This is perfectly reasonable under PH, when the null hypothesis concerns the HR, and the latter is a design parameter which is meaningful and independent of follow-up time. Many earlier trials, for example some in oncology with simpler research regimens, were reasonable candidates for PH and were possibly too small to detect important non-PH except in rare cases. Today, treatments are more complex, sample sizes are often large and follow-up is sometimes of necessity long (e.g. in screening trials for relatively rare conditions). Consequently, the chance of encountering non-PH is much larger than before. It may be argued that what is needed is a resilient test and, not necessarily coherent with it, relevant measures to help describe and interpret the treatment effect. Of the tests we have studied here, several are constructed from more than one component and therefore have no obvious associated estimate.

In the case of non-PH (and, arguably, even of PH), no single summary measure can adequately capture the treatment effect. One is left with careful inspection of the estimated survival curves in order to judge the clinically relevant nature and magnitude of the treatment effect. Investigation of the related topics of estimation and interpretation is beyond the scope of the present paper. We shall discuss these topics in a later paper.

We also note that some people object to tests that place more weight at certain times compared with others, for example by placing more weight on a later event. Such weighting schemes may imply that having a late event is worse than having an early event. When the survival curves cross, it can even happen that LRL rejects in favour of one treatment arm and LRE in favour of the other arm. Our view is that we are testing whether the two survival curves are equal. If we conclude that they are not, and the curves cross, the preferred treatment will depend on individual preferences regarding the trade-off between early versus later risks.

A key question potential users will ask is how high a cost (i.e. increase in sample size) is incurred under PH when using a test other than the standard logrank. Our results on power deficit (see Fig. 12) do address this issue, but further simulations, for example with different control-arm survival distributions and event rates, would certainly be desirable.

Where do we go from here? The only test that has been extensively researched, implemented, validated and used in a multitude of trials is the logrank. Furthermore, monitoring trial maturity and hence determining when the trial is ready to analyse is straightforward, requiring only the cumulative number of events. Under non-PH and using a different test, how best to assess maturity is an open issue. Further experience with the power of a test in different non-PH situations is needed.

How would a test be used in practice? Stata software is (or will soon be) freely available to perform all nine tests investigated here, and power/sample size calculations have been implemented for some of the non-standard tests, e.g. those in [25, 26] for the combined test (C), and will soon be made available for the preferred test, Ka2. Clearly, a preferred test would have to be specified up front in the study protocol for use in the sample size calculations and in the primary analysis. We stress the need to avoid defective statistical practice, such as performing a logrank test first and finding it to be ‘nearly’ significant, followed up by (say) a combined test to try to obtain more power and ‘achieve’ the magic *p*<0.05. How to implement appropriate guidance (e.g. stopping rules) for benefit or lack of benefit at interim analyses when using an alternative test also requires investigation.

## Conclusions

On present evidence, our test of choice is VWLR2. The recommendation assumes ignorance of the type of treatment effect to be expected. Several tests performed well when the correct type of treatment effect was assumed: SupLR, C, VWLR2, J, LRE and WC with an early effect; LRL, VWLR, VWLR2, WC and J with a late effect; and LR with a PH or near-PH effect. A low control-arm event rate reduced the power of weighted logrank tests targeting early effects. Test size was somewhat inflated with a high event rate and less than about 100 events in the dataset. The results must be regarded as initial. Further investigation of test characteristics with different types of non-proportional hazards of the treatment effect may be required.

## Data Availability

The datasets used to provide control-arm survival distributions as the basis of simulation studies are available from the corresponding author on reasonable request.

## Abbreviations

C: Combined
HR: Hazard ratio
J: Joint
LR: Logrank
LRE: Logrank (early)
LRL: Logrank (late)
MRC: Medical Research Council
Non-PH: Non-proportional hazards
PH: Proportional hazards
RCT: Randomized controlled trial
SupLR: Supremum logrank
UCL: University College London
VWLR2: Modified versatile weighted logrank
VWLR: Versatile weighted logrank
WC: Weighted combined
